# Response to Letter to the Editor regarding Factors associated with oral anticoagulant prescription status among patients with a new diagnosis of atrial fibrillation

**DOI:** 10.1002/clc.24122

**Published:** 2023-08-15

**Authors:** Evan Manning, Michael Ho, Amneet Sandhu

**Affiliations:** ^1^ Internal Medicine Residency Training Program University of Colorado Anschutz Medical Campus Aurora Colorado USA; ^2^ Deparment of Medicine University of Colorado Anschutz Medical Campus Aurora Colorado USA

Response to Letter to the Editor

We appreciate the comments from Drs. Kataoka and Imamura in their Letter to the Editor regarding our recent publication Factors Associated with Oral Anticoagulant Prescription Status Among Patients with a New Diagnosis of Atrial Fibrillation. In this manuscript, we identified patients with newly diagnosed atrial fibrillation and described patient‐level factors associated with anticoagulant prescription status in the first 6 months following diagnosis.^1^

The study period of 2013–2018 was selected to utilize electronic health record administrative data, CHA_2_DS_2_‐VASc score, and pharmacy records that were validated by physician chart review as well as to avoid any confounding as a result of the COVID‐19 pandemic. The primary intent of our work was to explore factors associated with prescription of any appropriate oral anticoagulant (OAC) following a new diagnosis of atrial fibrillation. Trends of direct OAC (DOAC) versus vitamin K antagonist prescriptions following US Food and Drug Administration (FDA) approval of DOACs have been well described, indicating an uptick in the United States in 2012 following initial FDA approval in 2010.^2^, ^3^, ^4^

We focused our evaluation on patients with a CHA_2_DS_2_‐VASc score ≥2 based on the established guidelines for anticoagulation in atrial fibrillation during the study period.^5^ Distribution of CHA_2_DS_2_‐VASc scores from a subgroup analysis confirmed with chart review is provided in Figure 1. The average CHA_2_DS_2_‐VASc score in our study was 3.94 among those who received anticoagulation and 3.73 among those who did not receive anticoagulation. These scores were not statistically significant, suggesting that rates of timely OAC prescriptions were not driven by differential CHA_2_DS_2_‐VASc scores. Rates of OAC prescription in our study are also consistent with other work across the same time period, suggesting timely OAC prescription rates did not eclipse 50% even among patients with the highest CHA_2_DS_2_‐VASc scores. This further indicates that the magnitude of the CHA_2_DS_2_‐VASc score may not significantly impact the timeliness of OAC prescription.^6^, ^7^ Importantly, the study cited by Kataoka and colleagues stems from a database of patients within a universal healthcare system outside of the United States. Higher OAC prescription rates in the reference work may also be explained by geographical, cultural, and social differences in anticoagulation practice patterns given that the average CHA_2_DS_2_‐VASc score was lower in that study (2.8) than in our work.^8^

**Figure 1:**
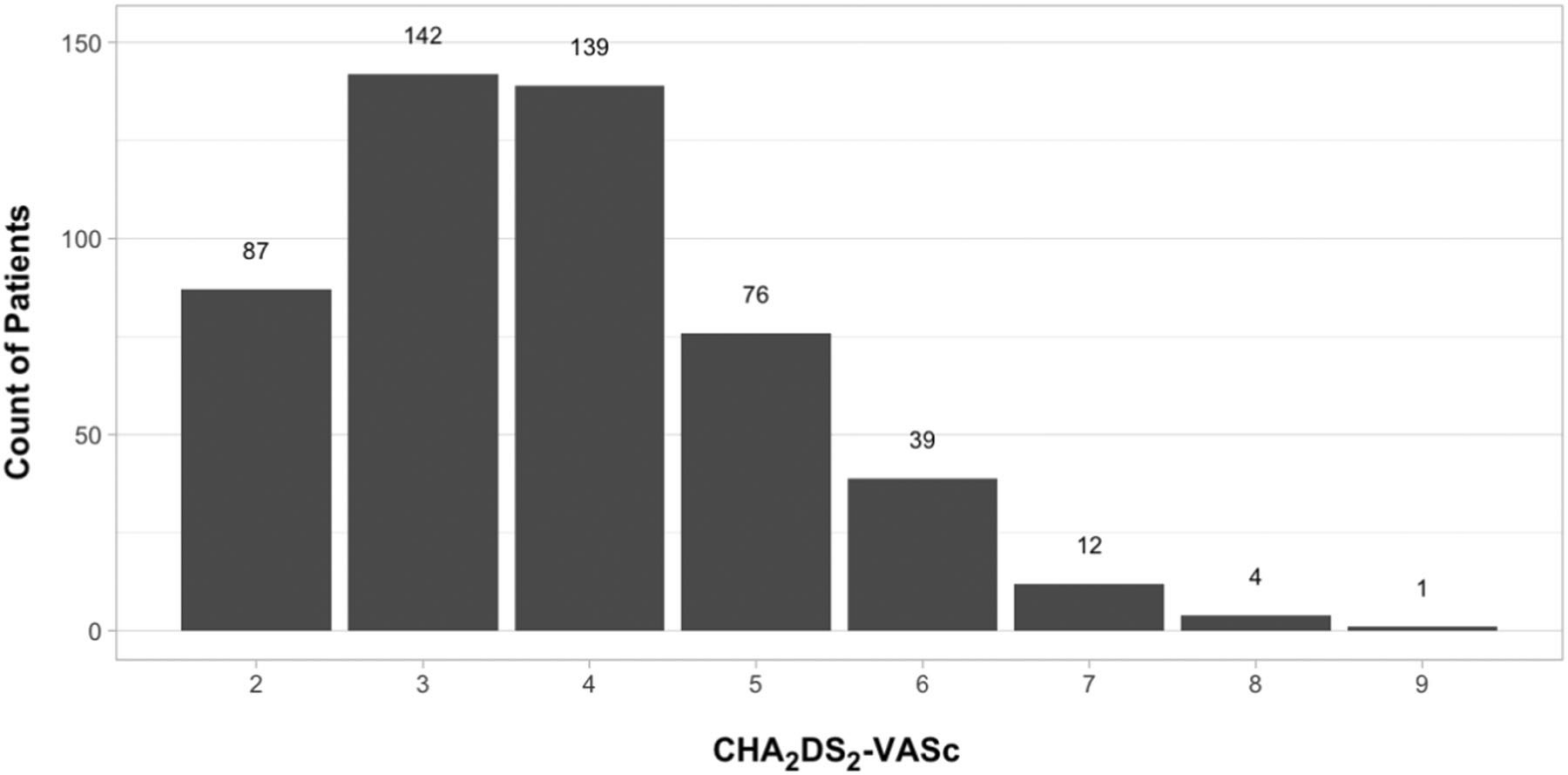
Distribution of CHA_2_DS_2_‐VASc scores as determined by physician chart review of a random cohort subgroup.

In response to the third comment regarding our finding of an inverse relationship of antiarrhythmic drug prescriptions and OAC prescriptions, we appreciate that the letter writers agree with our hypothesis that this association could be due to an incorrect practice habit in terms of rate versus rhythm control, which we highlighted in our discussion of the findings.^1^

Finally, data regarding the prevalence of left atrial appendage occlusion (LAAO) devices across our cohort was not available and outside the scope of our work. However, we expect the number of patients with LAAO devices in our cohort to be small and unlikely to explain the low OAC prescription rates. Currently, given payor guidelines, LAAO is most commonly reserved for patients who demonstrate recurrent failure of therapeutic anticoagulation which only rarely occurs during the first 6 months after atrial fibrillation diagnosis. During a similar time period, one study determined that the average of time between atrial fibrillation diagnosis and LAAO was 5.9 years and even in the PROTECT AF study, which specifically randomized patients for LAAO versus warfarin, the percentage of patients who received LAAO within 1 year of AF diagnosis was 14.9%.^9^, ^10^

## CONFLICT OF INTEREST STATEMENT

The authors declare no conflict of interest.

## ETHICS STATEMENT

This study was approved by the Colorado Multiple Institution Review Board under COMIRB protocol number 18‐2328. Subjects used in this research provided informed consent before the start of study activities.
